# Poly(ADP-ribose) polymerase inhibitors activate the p53 signaling pathway in neural stem/progenitor cells

**DOI:** 10.1186/s12868-016-0333-0

**Published:** 2017-01-17

**Authors:** Akiko Okuda, Suguru Kurokawa, Masanori Takehashi, Aika Maeda, Katsuya Fukuda, Yukari Kubo, Hyuma Nogusa, Tomoka Takatani-Nakase, Shujiro Okuda, Kunihiro Ueda, Seigo Tanaka

**Affiliations:** 1 Laboratory of Pathophysiology and Pharmacotherapeutics, Faculty of Pharmacy, Osaka Ohtani University, 3-11-1 Nishikiori-kita, Tondabayashi, Osaka 584-8540 Japan; 2 Bioinformatics, Niigata University Graduate School of Medical and Dental Sciences, 2-5274 Gakkochodori, Chuo-ku, Niigata, 951-8514 Japan; 3 Kobe Tokiwa University, 2-6-2 Otanicho, Nagata-ku, Kobe, Hyogo 653-0838 Japan; 4 Niigata University Graduate School of Health Sciences, 2-746 Asahimachidori, Chuo-ku, Niigata, 951-8518 Japan; 5 Department of Pharmaceutics, School of Pharmacy and Pharmaceutical Sciences, Mukogawa Women’s University, 11-68 Koshien-kyubancho, Nishinomiya, Hyogo 663-8179 Japan

**Keywords:** Poly(ADP-ribosyl)ation, Poly(ADP-ribose) polymerase, Neural stem/progenitor cells, p53, Cell cycle, Apoptosis

## Abstract

**Background:**

Poly(ADP-ribose) polymerase 1 (PARP-1), which catalyzes poly(ADP-ribosyl)ation of proteins by using NAD^+^ as a substrate, plays a key role in several nuclear events, including DNA repair, replication, and transcription. Recently, PARP-1 was reported to participate in the somatic cell reprogramming process. Previously, we revealed a role for PARP-1 in the induction of neural apoptosis in a cellular model of cerebral ischemia and suggested the possible use of PARP inhibitors as a new therapeutic intervention. In the present study, we examined the effects of PARP inhibitors on neural stem/progenitor cells (NSPCs) of the mouse brain.

**Results:**

PARP-1 was more abundant and demonstrated higher activity in NSPCs than in mouse embryonic fibroblasts. Treatment with PARP inhibitors suppressed the formation of neurospheres by NSPCs through the suppression of cell cycle progression and the induction of apoptosis. In order to identify the genes responsible for these effects, we investigated gene expression profiles by microarray analyses and found that several genes in the p53 signaling pathway were upregulated, including *Cdkn1a*, which is critical for cell cycle control, and *Fas*, *Pidd*, *Pmaip1*, and *Bbc3*, which are principal factors in the apoptosis pathway. Inhibition of poly(ADP-ribosyl)ation increased the levels of p53 protein, but not p53 mRNA, and enhanced the phosphorylation of p53 at Ser18. Experiments with specific inhibitors and also shRNA demonstrated that PARP-1, but not PARP-2, has a role in the regulation of p53. The effects of PARP inhibitors on NSPCs were not observed in *Trp53* ^−/−^ NSPCs, suggesting a key role for p53 in these events.

**Conclusions:**

On the basis of the finding that PARP inhibitors facilitated the p53 signaling pathway, we propose that poly(ADP-ribosyl)ation contributes to the proliferation and self-renewal of NSPCs through the suppression of p53 activation.

## Background

Poly(ADP-ribose) polymerase-1 (PARP-1) and PARP-2 belong to the PARP family, which consists of 17 predicted members that share a catalytic domain homologous to that of PARP-1 [1, 2]. These enzymes use NAD^+^ as a substrate, synthesize ADP-ribose molecules, and transfer them onto the glutamate, aspartate, or lysine residues of acceptor proteins. Poly(ADP-ribosyl)ation regulates nuclear functions and responses that include DNA repair, replication, transcription, and chromatin modification. After exposure to genotoxic chemicals, such as DNA alkylating agents, PARP-1 binds to the DNA strand breaks, resulting in a change of conformation and increase of its enzymatic activity by 10–500 fold [3–5]. The modified acceptor proteins, including histones and PARP-1 itself, greatly change their size by harboring up to several hundred ADP-ribose residues [2, 6]. The polyanionic structure thus formed counteracts the inhibitory effect of histones on DNA ligase. Conversely, excessive activation of PARP-1 and depletion of NAD^+^ after severe DNA damage cause cell death by ATP depletion or an “energy crisis” [7, 8]. Previously, we reported a principal role for PARP-1 in the induction of mitochondrial impairment that ultimately leads to neuronal apoptosis after cerebral ischemia [9], indicating that PARP inhibitors could be a good therapeutic intervention for cerebral infarction.

PARP inhibitors such as 3-aminobenzamide (3AB) interact with the nicotinamide pocket of PARP-1, which is a highly conserved region in the catalytic domain of PARPs, and act as competitors of NAD^+^ [10, 11]. Therefore, these inhibitors can suppress the activity of various PARPs with a homogeneous catalytic domain. More recently, however, several PARP inhibitors selective for PARP-1 or PARP-2 have been developed to study their specific function or potential therapeutic application [12]. As little is known about the effects of PARP inhibitors on somatic stem cells, these effects should be taken into consideration, particularly for their clinical use.

In the adult human and rodent brain, neural stem/progenitor cells (NSPCs) exist in the subventricular zone of the lateral ventricles and propagate to the olfactory bulb [13, 14]. NSPCs are also present in the subgranular zone of the hippocampal dentate gyrus and possibly contribute to spatial memory formation and cognition [15]. In these regions, neurogenesis occurs even in physiological conditions. However, under various types of brain injury, such as stroke, epileptic seizures, and trauma, the generation and proliferation of neural precursor cells are induced both in the subgranular and subventricular zones. The majority of neurons generated in the subventricular zone migrate toward the lesion site to replace damaged neurons and induce neural regeneration [16].

Mutation of the *p53* gene is observed frequently in cancer [17]. The function of p53 as a tumor suppressor depends principally on its ability to suppress cellular proliferation that would otherwise form tumor tissue. Activation of p53 induces cell cycle arrest and apoptosis [18, 19]. These functions of p53 result from its role as a transcription factor [20, 21]. Among the identified p53-target genes, p21 plays a critical role in the induction of cell cycle arrest [22, 23]. p21 is a cyclin-dependent kinase inhibitor that induces both the G1 and G2 cell cycle arrest observed after p53 activation [24–26]. Conversely, p53 induces apoptosis by activating some genes that participate in the apoptotic response. Furthermore, p53 plays a critical role in preventing the reprogramming of cells carrying various types of DNA damage [27]. Silencing of p53 significantly enhances the efficiency of the reprogramming of human somatic cells [28].

In the present study, we investigated the effects of PARP inhibitors on NSPCs in the adult brain and found two different effects, i.e., suppression of cell cycle progression and induction of apoptosis. Interestingly, both effects are mediated by the activation of p53. It is worthy of special mention that more poly(ADP-ribosyl)ated proteins existed in NSPCs than in mouse embryonic fibroblasts (MEFs). On the basis of these results, PARP, or poly(ADP-ribosyl)ation, could play a principal role in the maintenance of NSPC multipotency through the suppression of p53 function.

## Methods

### Separation and passage of NSPCs

All experimental protocols conformed to the Fundamental Guidelines for Proper Conduct of Animal Experiment and Related Activities in Academic Research Institutions under the jurisdiction of the Ministry of Education, Culture, Sports, Science, and Technology, Japan, and all experiments were approved by the Animal Experiment Committee of Osaka Ohtani University (No. 1012). NSPCs were obtained from Slc:ICR mouse embryos (embryonic day 13.5) as described previously [29–31]. The cells were dissociated and suspended at a density of 2.0 × 10^6^ cells in 100-mm dishes in 1× Dulbecco’s modified Eagle’s medium (DMEM)/F-12 neurosphere medium supplemented with B-27 (Gibco), 20 ng/mL human recombinant epidermal growth factor (EGF) (PeproTech), and 20 ng/mL human recombinant fibroblast growth factor (FGF)-basic (PeproTech). The culture medium was changed every other day and the cells were dissociated by using StemPro Accutase (Life Technologies) every 4 days. The cells were passaged 3–5 times. Untreated bacterial-grade culture dishes were used for suspension cultures, whereas dishes coated with poly-l-ornithine and fibronectin were used for monolayer cultures.

### Trp53 deficient mice

*Trp53*-heterozygous mice (accession no. CDB0001K) [32] were obtained from the RIKEN BioResource Center. Genotyping for the *Trp53* allele was performed by polymerase chain reaction (PCR) with primer 1 (5′-gttatgcatccatacagtaca-3′) and primer 2 (5′-caggatatcttctggaggaag-3′).

### PARP inhibitors

*N*-(6-oxo-5,6-dihydro-phenanthridin-2-yl)-*N,N*-dimethylacetamide (PJ34; Calbiochem), 1,5-isoquinolinediol (DHIQ; Santa Cruz Biotechnology), 3AB (Sigma), DR2313 (Wako Chemical), and UPF1069 (Wako Chemical) were used as PARP inhibitors.

### Immunocytochemistry

NSPCs were seeded at 5.0 × 10^4^ cells per well in 8-well poly-l-ornithine- and fibronectin-coated Lab-Tek II Chamber Slides (Nalge Nunc). They were incubated for 6 days with or without 20 μM PJ34 and the medium was changed every other day. Conversely, cells for the positive controls of neurons, astrocytes, and oligodendrocytes were incubated for 1 day in neural stem cell medium and then the medium was changed to 1× DMEM/F-12 supplemented with B-27 for differentiation and incubated for 6 days. The cells were fixed in acetone/methanol for 2 min. The antibodies to detect the following antigens were used for immunocytochemistry: nestin (sc-20978, 1:25; Santa Cruz Biotechnology or MAB353, 1:200; Chemicon), beta-III tubulin (MAB1195, 1:100; R&D Systems), GFAP (Z0334, 1:500; Dako Cytomation), CNPase (MAB326, 1:200; Chemicon), p21 (sc-53870, 1:100; Santa Cruz Biotechnology), p53 (2524, 1:100; Cell Signaling), and phospho-p53 (Ser18) (9284, 1:50; Cell Signaling). Alexa Fluor dye-conjugated secondary antibodies of donkey anti-mouse IgG-Alexa Fluor 488 (A21202, 1:500; Molecular Probes) and goat anti-rabbit IgG-Alexa Fluor 568 (A11036, 1:500; Molecular Probes) were used for detection. Nuclear staining was performed using 1 nM 4′, 6-diamidino-2-phenylindole (17514; ABD Bioquest). Cellular fluorescence images were acquired using a confocal laser scanning microscope (LSM 510; Carl Zeiss).

### MTS assay

NSPCs were seeded at 1.0 × 10^4^ cells per well in 96-well microplates coated with poly-l-ornithine and fibronectin. For 3-(4,5-dimethylthiazol-2-yl)-5-(3-carboxymethoxyphenyl)-2-(4-sulfophenyl)-2H-tetrazolium, inner salt (MTS) assay, a CellTiter 96 AQueous One Solution Cell Proliferation Assay kit (Promega) was used following the manufacturer’s instruction. Briefly, at 1 h before each of the desired time points, 20 µL MTS reagent were added to each well and the cells were incubated at 37 °C for 1 h. Absorbance was detected at 490 nm using a Microplate Reader (Model 680; Bio-Rad). All experiments were repeated 3 times.

### Gene expression profiling and data processing

Total RNA was extracted from NSPCs with or without treatment with 20 μM PJ34 by using an RNeasy Plus Mini Kit (QIAGEN). Microarray hybridizations were performed at Hokkaido System Science Co., Ltd. according to the manufacturer’s protocol using the workflow for Agilent SurePrint G3 Mouse GE (8 × 60 K) microarrays. Each total RNA was prepared independently twice and analyzed for 2 biological replicates. These data were deposited in the Gene Expression Omnibus (GEO) at NCBI (www.ncbi.nlm.nih.gov/geo/) (accession number GSE69038). Differential expression analysis was performed using the limma package [33]. A linear model was fitted to each gene, and empirical Bayes moderated *t*-statistics were used to assess differences in expression. The false discovery rate (FDR) adjusted *p* value was estimated using the Storey’s *q*-value method [34], and statistical significance for differential expression was set to *q* value <0.05 and *p* value <0.05, coupled with a minimal difference of absolute fold change >2. Genes reaching statistical significance were mapped on pathways by using the Kyoto Encyclopedia of Genes and Genomes (KEGG) database [35]. The number of genes in each KEGG pathway category was counted using the KEGG Orthology (KO) identifier. Subsequently, significantly enriched KEGG pathway categories were extracted based on *p* value <0.0001 and *q* value <0.01 by Fisher’s exact test, which was performed by using R (http://www.r-project.org/).

### Total RNA preparation and RT-PCR

Cells rinsed with phosphate-buffered saline (PBS) were treated with a NucleoSpin RNA Plus Kit (Macherey–Nagel). Total RNA was isolated according to the manufacturer’s protocol. RNA concentration was determined by measurement of *A*_260_. cDNA was made from total RNA using a ReverTra Ace Kit (Toyobo) with 0.5 μg total RNA per 25-μL reaction following the manufacturer’s instructions. Quantitative PCR for gene expression was performed with 2 μL diluted cDNA using KAPA SYBR Fast qPCR Master Mix (KAPA Biosystems) with specific primers (500 nM) in a total reaction volume of 5 μL. CFX96 Touch Real-time PCR System and CFX Manager software V3.1 (Bio-Rad Laboratories) were used to collect and analyze data. Three replicates of each sample were amplified. Relative quantitation of RNA levels was determined by comparative CT reactions (ΔΔC_T_ analysis). Primers for the amplification of mouse *Parp1*, *Parp2*, and glyceraldehyde 3-phosphate dehydrogenase (*Gapdh*) (Table 1) were used. *Gapdh* served as the endogenous control. The quantity of target mRNA in each knock-down cell was expressed in arbitrary units (relative quantitation).

**Table 1:** Primer sequences for RT-PCR gene expression analysis

Gene	Sense primer (5′–3′)	Anti-sense primer (5′–3′)	Cycles	Product (bp)
*Parp1*	TCGATGGGAAAGTCCCACAC	CATTCTGAGCCTTGAGGGCC	28	600
*Parp2*	GACAATCGAGACTCTGTGAA	AGACTGGTAACCGGCCTTGA	28	600
*Gapdh*	ACCACAGTCCATGCCATCAC	TCCACCACCCTGTTGCTGTA	28	452
*Cdkn1a*	GTGATTGCGATGCGCTCATG	TCTCTTGCAGAAGACCAATC	26	387
*Fas*	ATGCTGTGGATCTGGGCTGT	GTTTTCAGGTTGGCATGGTT	28	190
*Pidd*	ATGGCTGCAGTGTTGGAGGG	CTCTGAGAGATGGTTGTGAG	28	500
*Tnfrsf10b*	ATGGAGCCTCCAGGACCCAG	GAGCTCCAATCAGCAGCACT	28	643
*Pmaip1*	ATGCCCGGGAGAAAGGCGCG	GGTTACTAAATTGAAGAGCT	28	308
*Bbc3*	CCTCAGCCCTCCCTGTCACCAG	GGGTGAGGGTCGGTGTCGAT	35	232
*Perp*	ATGCTGCGCTGCGGCCTGGC	GGAACAACCAATCAAGATGA	28	500
*Ccng1*	ATGATAGAAGTACTGACAAC	GGTGTCGTGAACGAGTGAAT	24	500
*Trp53*	GGAGACATTTTCAGGCTTATGG	AGAAGGGACAAAAGATGACAGG	28	232

### Suppression of gene expression by shRNA

*Parp*-knockdown (KD) and pLKO.1 empty vectors for short hairpin RNA (shRNA)-expressing lentivirus were purchased from Open Biosystems. The RNAi consortium (TRC) numbers were as follows: TRCN0000071211 (*Parp1*-KD) and TRCN0000071216 (*Parp2*-KD). Lentivirus was produced by transient transfection of 293T cells using a Trans-Lentiviral shRNA Packaging Kit (Thermo Fisher Scientific). The virus supernatant was concentrated by using a Lenti-X™ Concentrator (Takara Bio). For virus infection, NSPCs were incubated with the concentrated virus supernatant in a 6-well plate and centrifuged at 1200×*g* for 1 h at 32 °C. The virus medium was removed at 6 h after infection and replaced with fresh medium. The infected cells were selected with 800 ng/mL puromycin at 2 days after infection. Incubation was continued for an additional 2 days. Silencing was then assessed by measuring the levels of *Parp1* and *Parp2* mRNA using RT-PCR.

### Western blot analysis

NSPCs (2.0 × 10^6^ cells) were seeded in 100-mm dishes containing the PARP inhibitors or vehicle for 24 h. Scraped cells were collected and added to a sample buffer solution containing 2-mercaptoethanol (2×) for SDS-PAGE (Nacalai Tesque) and incubated at 95 °C for 5 min. Proteins were separated using Mini-PROTEAN TGX gels (Bio-Rad), transferred with the Trans-Blot Turbo system (Bio-Rad), and detected with antibodies against the following proteins: beta-actin (A1978, 1:100,000; Sigma), p21 (sc-53870, 1:1000; Santa Cruz Biotechnology), p53 (2524, 1:10,000; Cell Signaling), phospho-p53 (Ser18) (9284, 1:1000; Cell Signaling), cleaved caspase-3 (Asp175) (9661, 1:1000; Cell Signaling), cleaved caspase-8 (Asp387) (9429, 1:1000; Cell Signaling), caspase-9 (9504, 1:1000; Cell Signaling), poly(ADP-ribose) (4336-BPC-100, 1:5000; Trevigen), PARP-1 (MCA1522G, 1:5000; Serotec), ataxia telangiectasia mutated (ATM) (ab2618, 1:5000; Abcam), and ataxia telangiectasia and Rad3-related (ATR) (sc-1887, 1:5000; Santa Cruz Biotechnology). A peroxidase-linked secondary antibody (NA931V, 1:1000; GE Healthcare) was used for detection. Antibody-antigen complexes were visualized by ImmunoStar LD (Wako). The chemiluminescent blots were imaged with a ChemiDoc XRS + imager (Bio-Rad) and analyzed by ImageLab software version 2.0.1 (Bio-Rad). All antibodies were distilled in HIKARI signal enhancer (Nacalai Tesque). For caspase activation, as a positive control, the cells were treated with 10 ng/mL cycloheximide (Cell Signaling) for 24 h and 50 ng/mL mouse tumor necrosis factor-α (Cell Signaling) for 6 h.

### Cell cycle analysis

The cells were synchronized by the double-thymidine-block method as described previously, with minor modifications [36, 37]. 2.0 × 10^6^ cells were seeded in twenty five 100 mm plates. One plate was used for time 0 (pretreatment). One set of 12 plates was used for PJ34 treatment, and another set of 12 plates was used for control. They were subjected to the first treatment with 2 mM thymidine (Sigma) for 14 h, and then incubated in thymidine-free medium for 12 h, followed by the second treatment with 2 mM thymidine for 14 h to arrest the cell cycle at the G1/S boundary. The cells were washed twice with 1× DMEM/F-12 and then incubated in neurosphere medium and analyzed every 2 h. The nuclei of the treated cells were collected and stained with propidium iodide using a Cell Cycle TEST-PLUS DNA Staining Kit according to the manufacturer’s instructions (Becton–Dickinson). The DNA content of the stained nuclei was measured using a FACSCalibur Flow Cytometer (Becton–Dickinson). The results were analyzed by using ModFit LT 3.0 software (Verity Software House).

### Flow cytometric analysis of cell death

After incubation with 20 μM PJ34 for 24 h, the cells were collected by low-speed centrifugation, washed with ice-cold PBS, and resuspended in Annexin-V Binding Buffer (Becton–Dickinson). To detect Annexin-V-positive cells, the cells were incubated with Annexin-V (Becton–Dickinson) and 7-AAD (Becton–Dickinson) for 15 min at room temperature. The cells were analyzed with a FACSCalibur Flow Cytometer (Becton–Dickinson).

### Chromatin staining for the detection of apoptosis

NSPCs were incubated with or without the PARP inhibitors on poly-l-ornithine/fibronectin-coated Lab-Tek II Chamber Slides (Nalge Nunc) for 24 h. The cells were fixed with 4% paraformaldehyde in PBS for 10 min, followed by chromatin staining with 1 μg/mL Hoechst 33258 (Sigma) to detect morphological changes of nuclei associated with apoptosis.

### Precipitation of poly(ADP-ribosyl)ated proteins

Poly(ADP-ribosyl)ated proteins were isolated by using a highly specific Af1521 Macrodomain Poly(ADP-ribose) Affinity Resin Set (Tulip Biolabs). NSPCs were incubated with or without 20 μM PJ34 for 24 h. Proteins were extracted from 4.0 × 10^6^ cells using a lysis buffer (RIPA Buffer; Nacalai Tesque) and incubated with the affinity resin overnight at 4 °C. The resin-bound proteins were dissociated from the affinity resin by incubation in SDS-sample buffer at 95 °C for 10 min and analyzed by immunoblotting.

### Immunoprecipitation

NSPCs were incubated with or without 20 μM PJ34 for 24 h. Cell lysates were prepared from 4.0 × 10^6^ cells using RIPA buffer (Nacalai Tesque). An antibody against p53 (2524, 1:500; Cell Signaling), ATM (ab2618, 1:500; Abcam), or ATR (sc-1887, 1:500; Santa Cruz Biotechnology) was added to the cell lysates, and μMACS Protein A/G MicroBeads (Miltenyi Biotec) were added to magnetically label the immune complexes. Magnetically labeled proteins were collected by using μ Columns and μMACS Separator (Miltenyi Biotec), and analyzed by immunoblotting.

### Statistical analysis

The data were expressed as the mean value ± standard error of the mean (SEM). We used one-way ANOVA followed by Tukey’s post hoc test to analyze the differences among the three or more groups, and used Student’s *t* test for the differences between the two groups. Results were considered statistically significant at *p* < 0.05.

## Results

### Suppression of neurosphere formation by PARP inhibitors

**Fig. 1:**
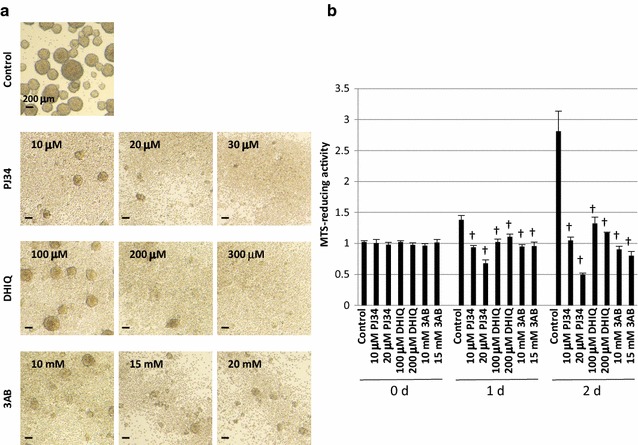
Suppression of neurosphere formation and cell viability of NSPCs by PARP inhibitors. **a** Multiple neurospheres were detectable after a 2-day incubation of NSPCs without a PARP inhibitor (control), while the number and size of neurospheres were much smaller in the presence of a PARP inhibitor (PJ34, DHIQ, or 3AB) in a dose-dependent manner. **b** Cell viability was determined by the MTS assay. The MTS-reduction activity of NSPCs was suppressed by the addition of a PARP inhibitor. Data shown in (**b**) are expressed as the ratio of the mean value of the control (*vehicle alone*) at time 0 (before incubation). Data represent the mean value ± SEM (n = 3). ^†^ *p* < 0.01 by comparison against control using one-way ANOVA followed by Tukey’s post hoc test

NSPCs from the mouse brain were incubated with or without the PARP inhibitors (3AB, DHIQ, or PJ34). Neurospheres were generated in the absence of the PARP inhibitors after incubation for 2 days, while neurosphere formation was suppressed by the PARP inhibitors in a concentration-dependent manner. Neurospheres were almost absent with 15 mM 3AB, 200 μM DHIQ, or 20 μM PJ34 (Fig. 1a). The concentrations of the 3 inhibitors required for these biological effects on NSPCs corresponded to their half maximal (50%) inhibitory concentrations (IC50s): IC50s of 3AB, DHIQ, and PJ34 were 33 μM, 390 nM, and 20 nM, respectively [38, 39]. An approximately 1000-fold higher concentration of the inhibitors, as compared with their IC50s, effectively suppressed neurosphere formation by NSPCs. We then evaluated the viability of NSPCs by measuring MTS-reducing activity, which was increased by 2.6-fold after culture for 2 days in the absence of the PARP inhibitors (Fig. 1b). This increase was significantly suppressed by the addition of the PARP inhibitors (3AB, DHIQ, or PJ34), indicating that exposure to the PARP inhibitors damages the viability of NSPCs.

### No induction of NSPC differentiation by a PARP inhibitor

**Fig. 2:**
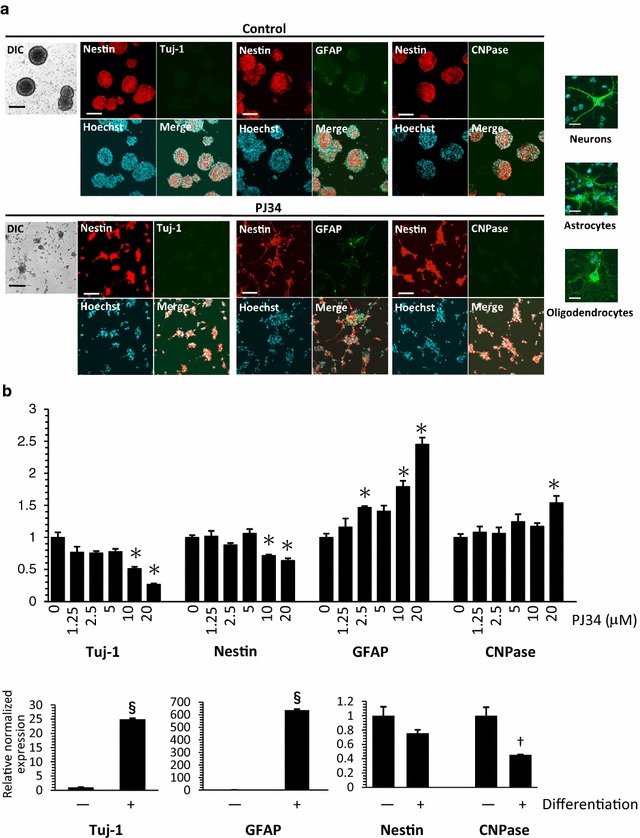
No induction of differentiation of NSPCs into neurons or glial cells by a PARP inhibitor. **a** The cells were triple-stained with Hoechst 33258 (nuclear staining), anti-nestin antibody (for NSPCs), and a cell-specific antibody: anti-Tuj-1 antibody (for neurons), anti-GFAP antibody (for astrocytes), or anti-CNPase antibody (for oligodendrocytes). Although the morphology of NSPCs was changed considerably after the addition of PJ34, the pattern of immunostaining was not changed. Differential interference contrast (DIC) images were on the left side. The positive controls for the antibodies used here are shown in the small images. **b** Quantitative mRNA analyses at 24 h after the addition of PJ34 (*shown in the upper row*) or 6 days after withdrawal of the growth factors EGF and FGF for differentiation into astrocytes (*shown in the lower row*) were performed for cell type-specific markers: Tuj-1 for neurons, nestin for NSPCs, GFAP for astrocytes, and CNPase for oligodendrocytes. Among these markers, GFAP was only upregulated by a few fold after the addition of 20 μM PJ34, while the expression of GFAP increased by more than 600-fold after in vitro differentiation of NSPCs into astrocytes. Data shown in (**b**) are expressed as the ratio of the mean value of the control (*vehicle alone*). Data represent the mean value ± SEM (n = 3).*p < 0.05 (*in the upper row*) by comparison against control using one-way ANOVA followed by Tukey’s post hoc test. ^†^ *p* < 0.01 and ^§^ *p* < 0.001 (*in the lower row*) by comparison against control using Student’s *t* test. Scale bars in (**a**), 100μm

While the PARP inhibitors suppressed NSPC neurosphere formation, a small population of NSPCs attached to the bottom of the culture dish and extended projections, suggesting their differentiation into neurons or glial cells (Fig. 2a). We examined the phenotype of these cells by immunostaining for several cell type-specific markers: nestin for NSPCs, Tuj-1 for neurons, GFAP for astrocytes, and CNPase for oligodendrocytes. Tuj-1 or CNPase was undetectable after incubation with or without PJ34 for 6 days, indicating no differentiation into neurons or oligodendrocytes, respectively, by this PARP inhibitor. Conversely, GFAP was detectable in a subpopulation of NSPCs both in the presence and absence of PJ34, indicating that NSPCs could be differentiated into astrocytes under the present experimental conditions regardless of their exposure to this PARP inhibitor. The ratio of nestin-positive cells appeared not to be changed by PJ34, suggesting no effects of this PARP inhibitor on NSPC differentiation. These morphological changes were supported by quantitative mRNA analysis of cell type-specific markers (Fig. 2b). Among these markers, GFAP was only upregulated by a few fold after the addition of 20 μM PJ34. Conversely, the expression of GFAP increased by more than 600-fold after in vitro differentiation of NSPCs into astrocytes. Most of the cells stopped proliferating and became apoptotic, while only a small number of cells, which attached to the bottom of the dishes, morphologically changed and extended projections. It appears that the cells that attached, or survived, differentiated into astrocytes, which likely caused a weak but significant upregulation of GFAP mRNA.

### Suppression of cell cycle progression by a PARP inhibitor

**Fig. 3:**
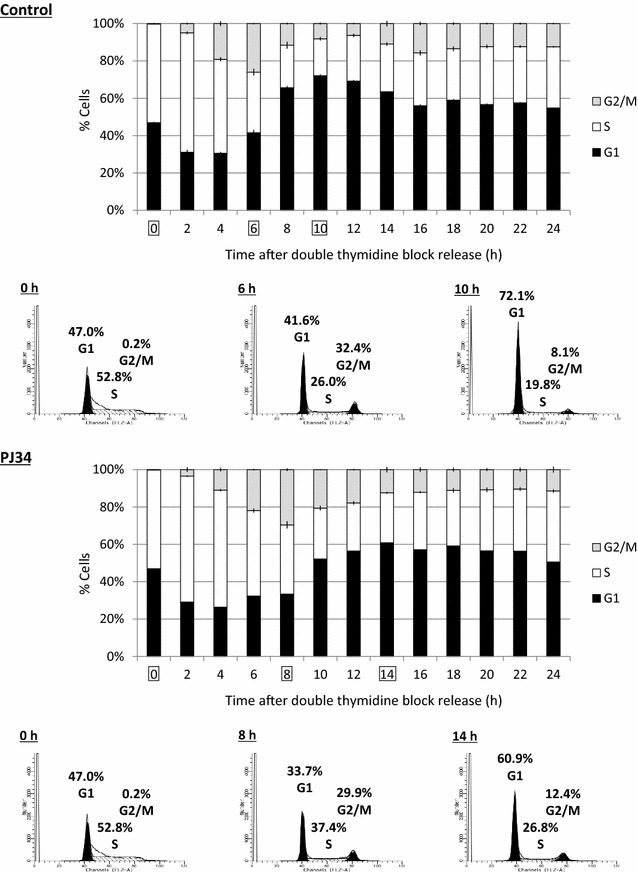
Suppression of cell cycle progression of NSPCs by PJ34. Progression of the cell cycle with or without PJ34 was analyzed by thymidine incorporation, followed by flow cytometry. The ratios of cells in the G1, S, and G2/M phases are illustrated every 2 h after the onset of the S phase. The results of flow cytometry at the peak of the G2/M or G1 phase are shown. Data represent the mean value ± SEM (n = 3)

We then assessed cell cycle progression in NSPCs by thymidine incorporation. S phase cells were obtained by using a double thymidine block that arrests the cells at the G1/S boundary. The removal of thymidine by replacement with normal medium, with or without the PARP inhibitor PJ34, induced the onset of the S phase. Every 2 h from the onset of DNA synthesis, the cell cycle was analyzed by flow cytometry (Fig. 3). The proportion of cells in the G2/M phase reached the maximum value at 6 h from the onset of the S phase in the absence of PJ34, while it took 8 h in the presence of PJ34. The peak of the proportion of cells in the G1 phase occurred after 14 or 10 h with or without PJ34, respectively. These results suggest that treatment with PJ34 suppressed cell cycle progression in NSPCs at the S phase and/or G2/M phase.

### Induction of apoptosis in NSPCs by PARP inhibitors

**Fig. 4:**
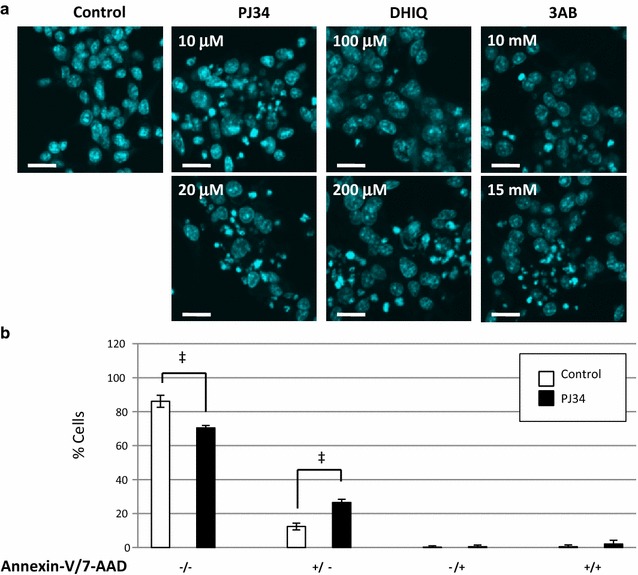
Induction of apoptosis in NSPCs by PARP inhibitors. **a** NSPCs were stained with Hoechst 33258 after a 24-h incubation with the PARP inhibitors. Cells with condensed chromatin, which is indicative of apoptosis, were detectable after the addition of PJ34, DHIQ, or 3AB. The ratio of apoptotic cells increased in a dose-dependent manner. **b** The rate of Annexin-V-positive cells after the addition of 20 μM PJ34 was analyzed by the use of flow cytometry staining with APC-conjugated Annexin-V and 7-AAD. Data represent the mean value ± SEM (n = 6). ^‡^ *p* < 0.005 by comparison against control using Student’s *t* test. Scale bars in (**a**), 20μm

The suppression of cell viability led us to examine whether the PARP inhibitors induce cell death, either necrosis or apoptosis, in NSPCs. Nuclear staining with Hoechst 33258 showed an intact or chromatin-condensed apoptotic pattern. Apoptotic cells prevailed after exposure to the PARP inhibitors (PJ34, DHIQ, or 3AB) in a concentration-dependent manner (Fig. 4a).

The pattern of cell death was then analyzed by the use of flow cytometry with APC-conjugated Annexin-V and 7-AAD. Annexin-V has high affinity for phosphatidylserine that is translocated from the inner to the outer leaflet of the plasma membrane during apoptosis. The cell membrane of live cells is impenetrable to 7-AAD, but 7-AAD readily permeates and stains dead cells. Annexin-V(+)/7-AAD(−) cells were considered to be at the early stage of apoptosis, whereas Annexin-V(+)/7-AAD(+) cells could be either necrotic or apoptotic at a later stage. Treatment with PJ34 significantly increased the proportion of apoptotic Annexin-V(+)/7-AAD(−) cells by 27% compared to 12% in the control cells and decreased that of intact Annexin-V(−)/7-AAD(−) cells by 71% compared to 87% in the control cells (Fig. 4b).

### Changes of mRNA expression profiles in the p53 signaling pathway by PARP inhibitors

**Fig. 5:**
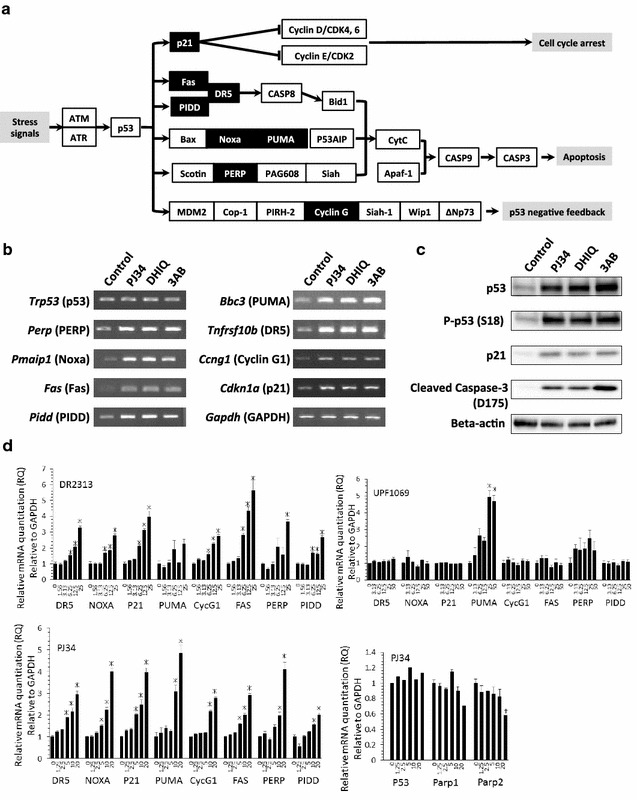
Change of mRNA expression profiles in the p53 signaling pathway. **a** The mRNA expression profiles were generated by microarray analysis. The genes, the expression level of which increased more than 2-fold after PARP inhibition, are indicated as a *black box* in the p53 signaling pathway published by the KEGG database. **b** The mRNA expression profiles shown in (**a**) were confirmed by RT-PCR. Several genes downstream to *Trp53* (p53) were upregulated after PARP inhibition, whereas *Trp53* itself was constant regardless of the presence of the PARP inhibitors. **c** Changes in the levels of p53 and phosphorylated p53 by the PARP inhibitors (PJ34, DHIQ, or 3AB) were observed in NSPCs. p21 protein as well as p21 mRNA, as shown in (**b**), were upregulated by all of the PARP inhibitors. Cleaved fragment of caspase-3 at Asp 175 (p17), which is a marker of apoptosis, was also increased by PARP inhibition. **d** Quantitative mRNA analyses after the addition of PJ34, DR2313 (PARP-1-specific inhibitor), or UPF1069 (PARP-2-specific inhibitor) were performed for the genes in the p53 signaling pathway. DR2313 as well as PJ34 upregulated the genes in the p53 signaling pathway, while UPF1069 did not change the expression of these genes, except *Bbc3* (PUMA). Data shown in (**d**) are expressed as the ratio of the mean value of the control (*vehicle alone*). Data represent the mean value ± SEM (n = 3). *p < 0.05 by comparison against control using one-way ANOVA followed by Tukey’s post hoc test

In order to identify the causative genes that are involved in the pathway leading to the suppression of cell cycle progression and induction of apoptosis, microarray analyses were carried out. The mRNA expression profiles in NSPCs were compared between cells in the presence and absence of PJ34. We found 142 genes upregulated and 83 genes downregulated by PJ34 with more than a twofold change (*p* < 0.05). In addition, several genes related to the p53 signaling pathway were significantly upregulated after the addition of PJ34, as shown by differential expression analysis (*p* value = 2.02E−8 and *q* value = 8.48E−7) (Table 2; Fig. 5a). The upregulation of *Cdkn1a* (p21), which plays a critical role in the control of the cell cycle, was more than twofold in the two analyses performed. Several genes in the pathway leading to apoptosis, including *Fas* (Fas), *Pidd* (PIDD; p53-induced death domain protein), *Pmaip1* (Noxa), and *Bbc3* (PUMA; p53 upregulated modulator of apoptosis), were also upregulated by more than 2-fold. Furthermore, *Ccng1* (cyclin G1), which is involved in p53 negative feedback, was also upregulated. These results from the microarray analyses were confirmed by RT-PCR (Fig. 5b), indicating that PJ34 and also the other PARP inhibitors, DHIQ and 3AB, activate the two pathways downstream of p53: one pathway leads to cell cycle arrest through the activation of p21, while the other induces apoptosis by some apoptosis-related factors. Interestingly, the RT-PCR results demonstrated that the amount of *p53* mRNA in NSPCs was not changed by the PARP inhibitors, whereas p53 protein was increased by the PARP inhibitors (Fig. 5c). This inconsistency indicated that the increase of p53 protein was not due to the overexpression of *Trp53* (p53), but to the suppression of p53 protein degradation. In this context, the phosphorylation of p53 at Ser 18, which inhibits the binding of Mdm2 and stabilizes p53 protein, was found to be increased by the PARP inhibitors. The amount of p21 protein was definitely increased by the PARP inhibitors, which is consistent with the upregulation of p21 mRNA expression. Furthermore, activation of the p53 signaling pathway to apoptosis resulted in the cleavage of pro-caspase-3.

**Table 2:** Statistically significant changed categories (control vs. PJ34 in wild-type or *Trp53* ^−/−^)

Gene name	Product	Probe name	Fold change	*p* value	*q* value
142 genes were up-regulated by PJ34 in wild-type NSPCs					
p53 signaling pathway					
*Bbc3*	PUMA (BCL2 binding component 3)	A_51_P248122	2.82	0.0136	0.0026
*Ccng1*	Cyclin G1	A_52_P612803	2.46	0.0240	0.0028
*Cdkn1a*	p21 (cyclin-dependent kinase inhibitor 1A)	A_51_P363947	2.99	0.0082	0.0023
*Fas*	Fas (TNF receptor superfamily member 6)	A_55_P2091676	3.50	0.0053	0.0018
*Lrdd*	PIDD (p53 induced death domain protein 1)	A_55_P2085485	2.24	0.0215	0.0028
*Perp*	PERP (p53 apoptosis effector)	A_51_P317941	5.03	0.0028	0.0016
*Pmaip1*	Noxa (phorbol-12-myristate-13-acetate-induced protein 1)	A_51_P477121	3.80	0.0144	0.0026
*Tnfrsf10b*	DR5 (tumor necrosis factor receptor superfamily, member 10b)	A_55_P2027836	3.23	0.0295	0.0029
83 genes were down-regulated by PJ34 in wild-type NSPCs					
n/a					
121 genes were up-regulated by PJ34 in *Trp53* ^−/−^ NSPCs					
n/a					
166 genes were down-regulated by PJ34 in *Trp53* ^−/−^ NSPCs					
n/a					

n/a indicates all categories’ *p*-values was more than 1E−04

In order to clarify which member of the PARP family is responsible for these effects of PJ34 on NSPCs, we used quantitative mRNA analysis to evaluate the effects of the inhibitors DR2313 and UPF1069, which are specific for PARP-1 and PARP-2, respectively (Fig. 5d). DR2313 upregulated the genes in the p53 signaling pathway in a similar way as PJ34, while UPF1069 did not change the expression of these genes, except *Bbc3* (PUMA). These findings confirmed PARP-1, but not PARP-2, as a target enzyme of PJ34 for its effects on the p53 signaling pathway in NSPCs.

### Effects of PARP-1 or PARP-2 knockdown on the p53 signaling pathway in NSPCs

**Fig. 6:**
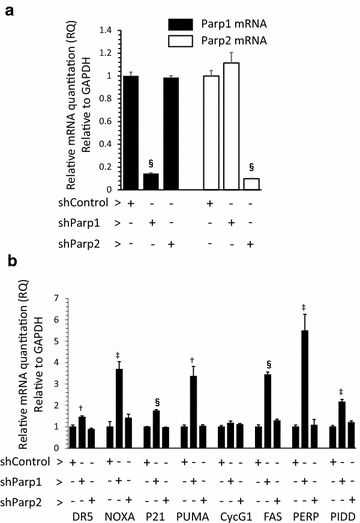
Effects of PARP-1 or PARP-2 knockdown on the p53 signaling pathway in NSPCs. Quantitative mRNA analyses after silencing *Parp1* or *Parp2* by shRNA-expressing lentivirus were performed for *Parp1* and *Parp2* (**a**) and the genes in the p53 signaling pathway (**b**). Genes in the p53 signaling pathway were upregulated by the suppression of *Parp1* expression, but not by *Parp2* suppression. Data shown in (**a**, **b**) are expressed as the ratio of the mean value of the sh Control group. Data represent the mean value ± SEM (n = 3). ^†^ *p* < 0.01, ^‡^ *p* < 0.005, and ^§^ *p* < 0.001 by comparison against control using Student’s *t* test

We then carried out silencing of *Parp1* or *Parp2* by infection with shRNA-expressing lentiviruses. *Parp1* or *Parp2* shRNA reduced the expression of the target gene to approximately 10% compared with control shRNA (Fig. 6a). Genes in the p53 signaling pathway were upregulated by the suppression of *Parp1* expression, but not by *Parp2* suppression (Fig. 6b). These findings were consistent with those obtained in the inhibitor experiments, and indicate that PARP-1, but not PARP-2, regulates the functions of p53 in NSPCs.

### Activation of p53 by PARP inhibitors

**Fig. 7:**
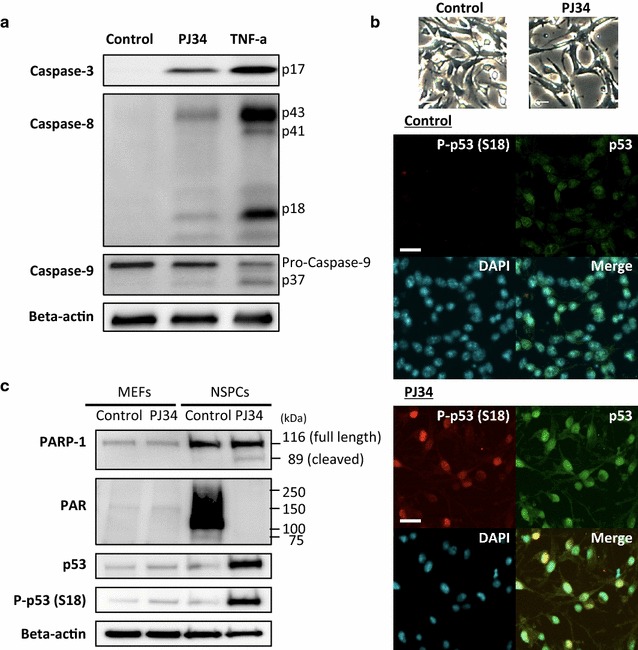
Higher amounts of p53 protein and phosphorylated p53 in NSPCs after PARP inhibition. **a** Cleaved fragments of caspase-3 (p17) and caspase-8 (p43/p41/p18) were clearly detectable by western blotting with specific antibodies. The cleaved fragment of pro-caspase-9 (p37) was also detectable, although the band density of this fragment was low. **b** Phase-contrast images were shown on the top. Higher amounts of p53 protein and phosphorylated p53 in NSPCs after PARP inhibition were also revealed by immunocytochemistry. Representative images from 4 separate experiments are shown. **c** Western blot analysis demonstrated that PARP-1 was abundant and activated in NSPCs in contrast to MEFs. Amounts of p53 protein and phosphorylated p53 at Ser 18 were remarkably increased by PJ34. Scale bars in (**b**), 20μm

The induction of apoptosis by the PARP inhibitors was demonstrated again by the detection of cleaved caspase fragments (Fig. 7a). One of the effector caspases, caspase-3, was activated, as revealed by the detection of the cleaved fragment (p17) of pro-caspase-3. Among the initiator caspases, caspase-8 and caspase-9 were also demonstrated to be activated by the detection of the cleaved fragments of the pro-caspases p43/p41/p18 and p37, respectively, although the band density of p37 was low.

The increased amount of total p53 and its phosphorylated form after the addition of PJ34 was revealed by immunostaining with antibodies against p53 and phosphorylated p53 (Ser18), respectively (Fig. 7b). Furthermore, NSPCs contained much higher levels of PARP-1 protein than MEFs and this enzyme was constitutively activated, as revealed by its automodification, in NSPCs (Fig. 7c). The 89-kDa fragment of PARP-1, which is generated by its cleavage by activated caspase-3, was also detectable. The increase of p53 protein and its phosphorylation in the presence of PJ34 were observed in NSPCs, but not in MEFs.

### Effects of a PARP inhibitor on Trp53^+/−^ and Trp53^−/−^ NSPCs

**Fig. 8:**
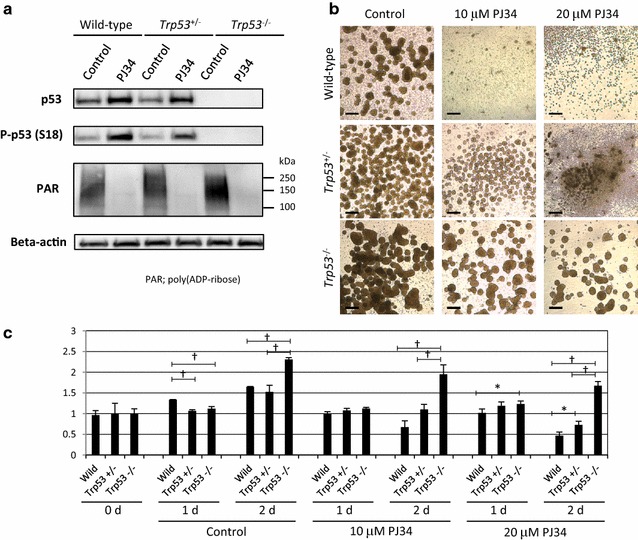
Effects of PJ34 on neurosphere formation and cell viability of *Trp53* ^+/−^ and *Trp53* ^−/−^ NSPCs. **a** Western blot analysis demonstrated that the amounts of p53 protein and phosphorylated p53 at Ser18 were increased by PJ34 in *Trp53* ^+/−^ NSPCs as well as wild-type NSPCs. Automodification of PARP-1 was detectable irrespective of *Trp53* genotype. **b** Neurospheres were detectable in wild-type NSPCs after a 2-day incubation in the absence of PJ34, but were scarcely detectable with 10 or 20 μM PJ34. In contrast, neurospheres were still detectable with 10 μM PJ34 in *Trp53* ^+/−^ NSPCs, and with 10 or 20 μM PJ34 in *Trp53* ^−/−^ NSPCs. **c** The increase of MTS-reduction activity of wild-type NSPCs was suppressed by 10 or 20 μM PJ34. No suppressive effect of PJ34 was observed in *Trp53* ^−/−^ NSPCs. Data represent the mean value ± SEM (n = 3). *p < 0.05 and ^†^ *p* < 0.01 by one-way ANOVA followed by Tukey’s post hoc test. Scale bars in (**b**), 50μm

In order to confirm a key role for p53 in the process under the regulation of poly(ADP-ribosyl)ation, we examined the effects of a PARP inhibitor on *Trp53* ^−/−^ as well as *Trp53* ^+/−^ NSPCs. PARP inhibition by PJ34 increased both total and phosphorylated p53 levels in wild-type and *Trp53* ^+/−^ NSPCs (Fig. 8a). *Trp53* ^−/−^ NSPCs formed larger neurospheres than *Trp53* ^+/−^ NSPCs, while *Trp53* ^+/−^ NSPCs generated a larger number of neurospheres than wild-type NSPCs (Fig. 8b). After incubation with 10 μM or 20 μM PJ34 for 2 days, only a few neurospheres were observed in wild-type NSPCs. In *Trp53* ^+/−^ NSPCs, neurospheres were still abundant in the presence of PJ34 at 10 μM, but not at 20 μM, although the neurospheres became smaller. Remarkably, even at a concentration of 20 μM, a substantial number of neurospheres were still present in *Trp53* ^−/−^ NSPCs.

Cell viability was evaluated by measuring MTS-reducing activity. It should be noted that *Trp53* ^−/−^ NSPCs showed higher activity than wild-type or *Trp53* ^+/−^ NSPCs in the absence of PJ34 (Fig. 8c). After the addition of 10 μM or 20 μM PJ34, the MTS-reducing activity of wild-type NSPCs was decreased. PJ34 also decreased the MTS-reducing activity of *Trp53* ^+/−^ NSPCs at a concentration of 20 μM, but not 10 μM, whereas that of *Trp53* ^−/−^ NSPCs was increased even after the addition of PJ34. These results clearly indicate that the effect of the PARP inhibitor PJ34 on the viability of NSPCs is mediated by p53.

In order to confirm the mRNA expression profiles in *Trp53* ^−/−^ NSPCs, mRNA levels were compared between the cells in the presence and absence of 20 μM PJ34 by microarray analyses. 121 genes were upregulated by and 166 genes were downregulated by PJ34. However, there was no significantly changed category (Table 2).

### Poly(ADP-ribosyl)ation of ATM and ATR

**Fig. 9:**
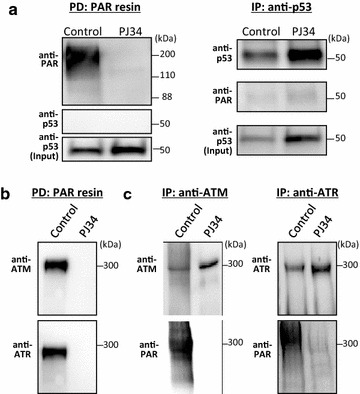
Poly(ADP-ribosyl)ation of ATM and ATR, but not p53. **a** Poly(ADP-ribosyl)ated proteins were isolated from cell lysates by highly specific macrodomain poly(ADP-ribose) affinity resin. The resin-bound proteins were eluted and analyzed by western blotting with an anti-p53 antibody. Immunoprecipitation was carried out using an anti-p53 antibody. The resulting immunocomplexes were subjected to western blot analysis with anti-p53 and anti-poly(ADP-ribose) antibodies. Poly(ADP-ribosyl)ated p53 was undetectable irrespective of PARP inhibition. **b** The same membrane on which the proteins eluted from poly(ADP-ribose) affinity resin were transferred was analyzed by western blotting with anti-ATM and anti-ATR antibodies, resulting in the detection of ATM and ATR. **c** Immunoprecipitation was performed using an anti-ATM or anti-ATR antibody. Immunocomplexes were subjected to western blot analysis with an anti-ATM or anti-ATR antibody as well as an anti-poly(ADP-ribose) antibody. Poly(ADP-ribosyl)ated ATM and ATR were detectable in the absence of PJ34

As demonstrated by the experiments using *Trp53* ^−/−^ NSPCs, p53 plays a key role in the regulation of cell viability by the PARP inhibitors. We then examined the molecular mechanism of p53 activation by the PARP inhibitors. We performed a pull-down assay using poly-ADP-ribose affinity resin, but were unable to isolate poly(ADP-ribosyl)ated p53 (Fig. 9a). An immunoprecipitation study using an anti-p53 antibody showed the increased quantity of p53 protein after exposure to the PARP inhibitor PJ34, while poly(ADP-ribosyl)ation of p53 was undetectable.

As both ATM and ATR regulate the stability and activation of p53 by phosphorylation at Ser18 [40], we examined whether PARP inhibition affects these 2 serine/threonine protein kinases. We performed a pull-down assay with poly-ADP-ribose affinity resin, and detected both ATM and ATR at their expected sizes using specific antibodies (Fig. 9b). Certainly, the addition of PJ34 inhibited the isolation of both kinases by poly-ADP-ribose affinity resin. Poly(ADP-ribosyl)ation of these two kinases was confirmed by immunoprecipitation with an anti-ATM or anti-ATR antibody followed by detection with an anti-poly(ADP-ribose) antibody (Fig. 9c). Together with the finding of PARP-1 activation in NSPCs (see Fig. 9c), both ATM and ATR were found to be constitutively poly(ADP-ribosyl)ated in these cells.

## Discussion

NSPCs are localized in specific areas in the adult brain such as the subgranular zone of the dentate gyrus of the hippocampus and subventricular area of the lateral ventricles [13, 14]. Once the brain is damaged by ischemia, NSPCs are activated and move to the infarct area [16]. Previously, we reported that PARP inhibitors could be a good therapeutic intervention for ischemic brain disorders through inhibition of apoptosis as well as necrosis of the affected neurons [9]. Lacza et al. [41] reported an improvement of the effectiveness of neural stem cell transplantation through the suppression of the ONOO^−^–PARP activation cascade by PARP inhibitor. However, administration of PARP inhibitors might influence NSPCs in the brain. In the present study, we demonstrated two types of effects of PARP inhibitors on NSPCs: suppression of cell cycle progression and induction of apoptosis. From a therapeutic point of view, these effects should be taken into consideration to determine the appropriate doses of PARP inhibitors.

The key molecule underlying these effects of the PARP inhibitors was proven to be p53, which restricts cellular growth by inducing cell cycle arrest (at the G1 and/or G2 phase) or apoptosis [42, 43]. Several factors that influence the decision between the two types of effect include the expression level of p53, the type of stress signal, and cell type. Under our experimental conditions for NSPCs, where the cells were not exposed to specific stresses, the expression level of p53 was consistent and its phosphorylation, or activation, was suppressed. After the addition of the PARP inhibitors, phosphorylation of p53 at Ser18 was enhanced, followed by the upregulation of p21, which is a potent cyclin-dependent kinase inhibitor that functions as a regulator of cell cycle progression at the G1 and S phase [44].

The PARP inhibitor PJ34 also upregulated other p53-dependent factors in the pathways to apoptosis, i.e., Fas, PIDD, DR5, and PERP in the extrinsic apoptotic pathway and Noxa and PUMA in the intrinsic apoptotic pathway [45]. The extrinsic pathway is mediated by particular death receptors that are members of the tumor necrosis factor receptor family, including Fas, DR5, and PERP, whose activation induces the formation of the death-inducing-signaling-complex, and then activation of the caspase cascade, including caspase-8 and caspase-3. In our study, both caspases were definitely activated after PARP inhibition, as revealed by the detection of the cleaved fragments of pro-caspases.

The intrinsic apoptotic pathway is activated in response to DNA damage and results in mitochondrial depolarization and release of cytochrome c into the cytoplasm [45]. Cytochrome c forms a complex “apoptosome” together with apoptotic protease-activating factor 1 and pro-caspase-9, followed by the activation of caspase-9 and then activation of other caspases such as caspase-3, -6, and -7 [46]. PJ34 induces the transcription of PUMA, also known as Bcl-2-binding component 3 (BBC3), which interacts with antiapoptotic Bcl-2 family members, leading to the formation of the free-type of Bax and/or Bak, which are then able to signal apoptosis to the mitochondria [47]. Intriguingly, a balance between PUMA and p21 reportedly determines the onset of cell cycle arrest, or death, in response to exogenous p53 expression. In our study, PJ34 also upregulated another p53 target gene, *Noxa*, which encodes a BH3-only protein and hence is considered to induce p53-mediated apoptosis in a manner similar to PUMA [45]. Thus, it appears that, in response to PARP inhibition, p53 activates the intrinsic apoptotic pathway by inducing the expression of at least two Bcl-2 pro-apoptotic family members including PUMA and Noxa. The fragment of pro-caspase-9, however, was only slightly detectable, indicating that the involvement of the intrinsic apoptotic pathway was limited.

The interaction of p53 with PARP-1 or poly(ADP-ribosyl)ation of p53 has been proposed by several investigators [48–51]. The DNA-binding domain of p53 contains some poly(ADP-ribose)-binding sites, which could interfere with its site-specific DNA-binding activity and block its transcriptional function [48, 49]. Alternatively, poly(ADP-ribose) could induce the upregulation of p53 by protecting the protein from proteolytic degradation. One of the poly(ADP-ribose) binding sites in p53 is located near a proteolytic cleavage site, which suggests that polymer binding might protect this sequence from proteolysis [51]. Interestingly, Kanai et al. [52] reported that poly(ADP-ribosyl)ation of p53 blocked the interaction between p53 and the nuclear export receptor Crm1, followed by the accumulation of p53 in the nucleus and activation of its transactivation function. These findings are not necessarily consistent with ours in which poly(ADP-ribosyl)ated p53 was undetectable. This inconsistency may be due to differences in the type of cell and stress utilized.

Some kinases, such as ATM and ATR, are reportedly responsible for the phosphorylation of p53 at Ser15 (human), i.e., Ser18 (mouse) [53, 54]. Phosphorylation at this site, as observed in our study, and also at Ser20 (human), could inhibit the binding of the p53-degrading enzyme Mdm2 (Hdm2 in humans), resulting in the stabilization of p53 [53]. Watanabe et al. [55] reported that after the induction of DNA double-strand breaks (DSBs) in MEFs by neocarzinostatin, PARP-1 negatively regulated ATM kinase activity and inhibited phosphorylation of p53 at Ser18. Kedar et al. [56] demonstrated that ATR interacted with PARP-1 after treatment with methyl methanesulfonate, but this interaction was not detectable after PARP inhibition. Under conditions of PARP inhibition in MEF culture, the cells accumulated in the S phase, probably due to ATR activation. They also demonstrated that ATR is a substrate for poly(ADP-ribosyl)ation by PARP-1 in vitro. These results suggest that poly(ADP-ribosyl)ation of ATM and/or ATR after DSBs inactivates these kinases, leading to the inhibition of p53 phosphorylation. In our experiments using NSPCs without induction of DSBs, both ATM and ATR were poly(ADP-ribosyl)ated or inactivated. The PARP inhibitors removed this modification and activated these kinases, resulting in increased phosphorylation of p53 at Ser18 and stabilization of this protein.

PARPs constitute a large family of as many as 17 proteins [1], and PARP-1 is an abundant nuclear protein and the founding member of the PARP family. As poly(ADP-ribose) is mainly synthesized by PARP-1 after DNA damage, PARP-2 was initially thought to be a “backup of PARP-1.” On the contrary, *Parp*-*2* ^−/−^ mice exhibit impaired spermatogenesis [57], adipogenesis [58], and thymocyte survival [59], although *Parp*-*1* ^−/−^ mice differentiate normally in these processes. Thus, PARP-2 might have different targets from PARP-1, suggesting that they could play specific biological functions. Interestingly, PARP-1/PARP-2 double knockout mice, which lack poly(ADP-ribosyl)ation, are embryonic lethal [60]. Therefore, both PARP-1 and PARP-2 are thought to conduct critical roles in embryonic development. Our study using specific inhibitors and also shRNAs suggested that PARP-1, but not PARP-2, plays a role in the regulation of p53 functions. In this study, PUMA, a pro-apoptotic protein located in the p53-signaling pathway, was upregulated after treatment with the PARP-2-specific inhibitor UPF1069, which may be due to an unknown function of UPF1069 besides PARP-2-specific inhibition. This possibility should be investigated in further experiments.

Recent reports have provided evidence that intracellular programs, including epigenetic modifications, transcription factors, and extracellular signals, such as various cytokines, are involved in the induction of NSPC differentiation [61]. PARP1 as well as Tet2 are responsible for epigenetic modifications during the reprogramming process [62]. PARP-1 expression was found to be enhanced both in embryonic stem cells and induced pluripotent stem (iPS) cells. PARP-1 activation plays a key role both in the induction of iPS cells and the maintenance of pluripotency [63]. Interestingly, several groups have reported that p53 suppresses iPS cell generation and that its molecular mechanism involves two types of ability of p53: induction of p21, resulting in a restriction of cell cycling, and induction of apoptosis [27, 28, 64]. On the basis of these findings and our predictive model, for the maintenance of NSPC multipotency as well as pluripotency of iPS cells, suppression of p53 function by PARP-1 might be required.

## Conclusions

**Fig. 10:**
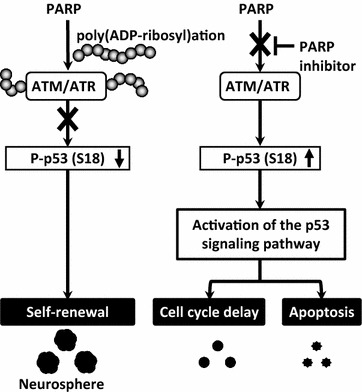
A model of cell fate decision by poly(ADP-ribosyl)ation in NSPCs. In normal conditions, both ATM and ATR are poly(ADP-ribosyl)ated and inactivated, leading to dephosphorylation of p53 at Ser18. The resulting inactivation of p53 promotes the self-renewal of NSPCs and neurosphere formation. Under PARP inhibition, ATM and/or ATR without poly(ADP-ribosyl)ation are activated, leading to the phosphorylation and activation of p53. The activated p53 signaling pathway delays cell cycle progression and induces apoptosis by different mechanisms

Our results indicate the possibility that PARP-1 activation or poly(ADP-ribosyl)ation contributes to the proliferation of NSPCs by advancing the cell cycle and suppressing apoptosis in addition to epigenetic modifications. Inactivation of ATM/ATR and the p53 pathway is suggested to be a mechanism that explains how PARP promotes proliferation and thus maintains NSPC multipotency (Fig. 10).
